# Blood-culture ordering practices in patients with a central line at an academic medical center–Iowa, 2020

**DOI:** 10.1017/ash.2022.112

**Published:** 2022-05-16

**Authors:** Elias Kovoor, Takaaki Kobayashi, Lorinda Sheeler, Alexandra Trannel, William Etienne, Oluchi Abosi, Stephanie Holley, Mary Kukla, Angie Dains, Kyle Jenn, Holly Meacham, Beth Hanna, Alexandre Marra, Meredith Parsons, Bradley Ford, Melanie Wellington, Daniel Diekema, Jorge Salinas

## Abstract

**Background:** The IDSA has a clinical definition for catheter-related bloodstream infection (CRBSI) that requires ≥1 set of blood cultures from the catheter and ≥1 set from a peripheral vein. However, because blood cultures obtained from a central line may represent contamination rather than true infection, many institutions discourage blood cultures from central lines. We describe blood culture ordering practices in patients with a central line. **Methods:** The University of Iowa Hospitals & Clinics is an academic medical center with 860 hospital beds. We retrospectively collected data for blood cultures obtained from adult patients (aged ≥18 years) in the emergency department or an inpatient unit during 2020. We focused on the first blood cultures obtained during each admission because they are usually obtained before antibiotic initiation and are the most important opportunity to diagnose bacteremia. We classified blood-culture orders as follows: CRBSI workup, non-CRBSI sepsis workup, or incomplete workup. We defined CRBSI workup as ≥1 blood culture from a central line and ≥1 peripheral blood culture (IDSA guidelines). We defined non-CRBSI sepsis workup as ≥2 peripheral blood cultures without cultures from a central line because providers might have suspected secondary bacteremia rather than CRBSI. We defined incomplete workup as any order that did not meet the CRBSI or non-CRBSI sepsis workup. This occurred when only 1 peripheral culture was obtained or when ≥1 central-line culture was obtained without peripheral cultures. **Results:** We included 1,150 patient admissions with 4,071 blood cultures. In total, 349 patient admissions with blood culture orders (30.4%) met CRBSI workup. 62.8% were deemed non-CRBSI sepsis workup, and 6.9% were deemed an incomplete workup. Stratified by location, ICUs had the highest percentage of orders with incomplete workups (8.8%), followed by wards (7.2%) and the emergency department (5.1%). In total, 204 patient admissions had ≥1 positive blood culture (17.7%). The most frequently isolated organisms were *Staphylococcus epidermidis* (n = 33, 16.2%), *Staphylococcus aureus* (n = 16, 7.8%), and *Escherichia coli* (n = 15, 7.4%) **Conclusions:** Analysis of blood culture data allowed us to identify units at our institute that were underperforming in terms of ordering the necessary blood cultures to diagnose CRBSI. Being familiar with CRBSI guidelines as well as decreasing inappropriate ordering will help lead to early and proper diagnosis of CRBSI which can reduce its morbidity, mortality, and cost.

**Funding:** None

**Disclosures:** None

